# Prognostic Value of Tumor Regression Grading in Patients Treated With Neoadjuvant Chemotherapy Plus Surgery for Gastric Cancer

**DOI:** 10.3389/fonc.2021.587856

**Published:** 2021-07-26

**Authors:** Jian-Wei Xie, Jun Lu, Bin-bin Xu, Chao-Hui Zheng, Ping Li, Jia-Bin Wang, Jian-Xian Lin, Qi-Yue Chen, Long-Long Cao, Mi Lin, Ru-Hong Tu, Ze-Ning Huang, Ju-Li Lin, Mark J. Truty, Chang-Ming Huang

**Affiliations:** ^1^Department of Gastric Surgery, Fujian Medical University Union Hospital, Fuzhou, China; ^2^Department of General Surgery, Fujian Medical University Union Hospital, Fuzhou, China; ^3^Key Laboratory of Ministry of Education of Gastrointestinal Cancer, Fujian Medical University, Fuzhou, China; ^4^Fujian Key Laboratory of Tumor Microbiology, Fujian Medical University, Fuzhou, China; ^5^Section of Hepatobiliary and Pancreatic Surgery, Division of Subspecialty General Surgery, Department of Surgery, Mayo Clinic, Rochester, MN, United States

**Keywords:** gastric cancer, neoadjuvant chemotherapy, tumor regression grading, signet-ring cell carcinoma (SRCC), recurrence-free survival (RFS) rate and overall survival (OS)

## Abstract

**Objective:**

To validate the prognostic value of tumor regression grading (TRG) and to explore the associated factors of TRG for advanced gastric cancer (AGC) with neoadjuvant chemotherapy (NACT) plus surgery.

**Methods:**

Two hundred forty-nine AGC patients treated with NACT followed by gastrectomy at the Mayo Clinic, USA and the Fujian Medical University Union Hospital, China between January 2000 and December 2016 were enrolled in this study. Cox regression was used to identify covariates associated with overall survival (OS) and recurrence-free survival (RFS). Logistic regression was used to reveal factors predicting tumor regression grading.

**Results:**

For patients with TRG 0-1, the 3- and 5-year OS rates were 85.2% and 74.5%, respectively, when compared to 56.1% and 44.1% in patients with TRG 2 and 28.2% and 23.0% in patients with TRG 3, respectively (p<0.001). TRGs were independent risk factors for OS. Similar findings were observed in RFS. Multivariable analysis revealed that an oxaliplatin-based regimen (p=0.017) was an independent predictor of TRG. The oxaliplatin-based regimen was superior to the nonoxaliplatin-based regimen for OS (38.4 months *vs* 19.5 months, respectively; p=0.01). Subgroup analyses by histological subtype indicated that the oxaliplatin-based regimen improved the OS in nonsignet ring cell carcinoma compared to the nonoxaliplatin-based regimen (53.7 months *vs* 19.5 months, respectively; p=0.011). However, similar findings were not observed in RFS.

**Conclusion:**

TRG was an independent factor of AGC treated with neoadjuvant chemotherapy plus surgery. Oxaliplatin-based neoadjuvant chemotherapy regimens improve tumor response and may have an overall survival benefit for patients with nonsignet ring cell carcinoma.

## Introduction

Gastric cancer is a leading cause of cancer-related mortality worldwide, with approximately 951,600 new cases diagnosed and 723,100 patients who succumb to the disease annually (1). The use of neoadjuvant chemotherapy in the treatment of patients with localized advanced gastric cancer has become more prevalent over the past ten years. Several advantages have been associated with this approach, including downgrading of the tumor, increasing the likelihood of achieving an R0 resection, and eradicating micrometastasis to reduce recurrence (2, 3). Neoadjuvant chemotherapy followed by gastrectomy could provide significant overall survival (OS) benefits over surgery alone (4).

Tumor regression grade (TRG) is a descriptive measurement defined as a histological response to neoadjuvant therapy and has shown prognostic value for digestive system tumors (5, 6). In 2003, TRG was first used by Becker et al. to evaluate the histological response in gastric cancer (7). TRG has been reported to be a predictor of survival in patients with gastric cancer in several studies (8, 9). A good tumor response rate significantly improved the OS and recurrence-free survival (RFS) (10). However, the factors associated with a better tumor response rate and an optimal neoadjuvant chemotherapy regimen that improved survival are uncertain.

Therefore, we investigated the role of TRG in neoadjuvant chemotherapy for gastric cancer and analyzed the factors affecting TRG to reveal the potential survival benefits of neoadjuvant chemotherapy for patients.

## Methods

### Patient Selection

Patients diagnosed with advanced clinical stage gastric cancer (more than clinical T2 category or clinical stage N1) were enrolled in this study. The exclusion criteria were as follows: multiple primary gastric cancer tumors, gastric cancer combined with other malignancies, history of radiotherapy or radiochemotherapy, types I and II esophagogastric junction tumors, and patients without tumor resection. Ultimately, a total of 249 patients were analyzed. Of these patients, 131 patients were submitted from the Fujian Medical University Union Hospital, and 118 patients were from the Mayo Clinic. Tumor staging was evaluated by the eighth edition of the Union for International Cancer Control (UICC) TNM classification system (11). Because the survival of patients with ypT0 and ypT1 was similar, we merged the patients with ypT0 into the ypT1 group.

### Variable and Definition

The RFS was calculated from surgery to the first event (i.e., local recurrence, distant recurrence, or death from any cause). The OS was calculated from when the disease was diagnosed to death or the final follow-up date in December 2017. According to the Japanese gastric cancer treatment guidelines (12), we divided the extent of lymph node dissection into D1 or D2. Similarly, we divided the resection margins into R0, R1 or R2. The score of tumor response regression was defined according to the recommendations of the College of American Pathologists as follows: 0=No viable cancer cells (complete response); 1=Minimal residual cancer with single cells or small groups of cancer cells (near complete response); 2=Residual cancer with evident tumor regression, which is more than single cells or small groups of cancer cells (partial response); and 3=Extensive residual cancer with no evident tumor regression (poor or no response) (13). The results were reviewed by two independent pathologists who were blinded to the clinical data. If the results of the same sample were discordant, then the pathologists would discuss to reach a final score.

### Treatment

Final decision to administer neoadjuvant chemotherapy, dose and cycles were made after careful discussion between the clinician and the patients. An oxaliplatin-based regimen was defined as a neoadjuvant chemotherapy regimen containing oxaliplatin. An epirubicin-based regimen was defined as a neoadjuvant chemotherapy regimen containing epirubicin. A total of 58 patients received the regimen containing both oxaliplatin and epirubicin. The median number of cycles of neoadjuvant chemotherapy was 3 (range 1-12).

Adjuvant chemotherapy: According to the patient’s wishes and their physical condition, fluoride-based adjuvant chemotherapy was recommended for most patients with pathological stage II and III disease in our center, as previously described. For patients who did not show histologic tumor regression before surgery, the adjuvant regimen was given different from the neoadjuvant regimen.

### Surgery

In general, resection of the gastric tumor with D2 lymphadenectomy was performed within 4 weeks after the last day of chemotherapy.

### Follow-up

Follow-up visits for both cohorts generally consist of clinic visits every 3 months for the first 2 years and every 6 months for years 3 to 5. Most routine patient follow-up appointments include a physical examination, laboratory tests, chest radiography, abdominal ultrasonography or CT, and an annual or biannual endoscopic examination for patients with a remnant stomach (14, 15).

### Statistical Analysis

Statistical analysis was performed using SPSS 20.0 software (SPSS, Chicago, IL). Intergroup comparisons for discrete variables were analyzed with the chi-squared test or Fisher’s exact test. The Kaplan–Meier method was used to calculate OS and RFS. A log-rank test was used to compare survival curves. The reverse Kaplan–Meier method was used to calculate the median follow-up time. A Cox regression model was used to calculate hazard ratios of ACT treatment. Ordinal regression was performed for relationships of covariates with TRG. Two-sided p-values < 0.05 were considered statistically significant.

## Results

### Patient Demographics and Neoadjuvant Treatment

The baseline characteristics of 249 patients are listed in **Table 1**. One hundred seventy-two (69%) patients were administered the oxaliplatin-based regimen, and 77 (31%) patients were administered the nonoxaliplatin-based regimen. The median number of cycles of neoadjuvant chemotherapy was 3 (range 1-12). Concerning histopathologic response evaluation, the TRG results for patients treated with neoadjuvant chemotherapy were as follows: TRG 0 (n = 12, 4.8%); TRG 1 (n = 35, 14.1%); TRG 2 (n = 74, 29.7%); and TRG 3 (n = 128, 51.4%). Because the survival of patients with TRG 0 and TRG 1 was similar, the cohort was divided into three groups: TRG 0 or TRG 1 (TRG 0-1), TRG 2, and TRG 3 (**Table 1**). The patient demographics among different TRG groups are listed in **Supplementary Table 1**.

**Table 1:** Baseline Characteristics.

Characteristic	Total (n=249)	China (N=131)	USA (N=118)	p value
Age(year)				0.376
<65	159	87	72	
≥65	90	44	46	
Sex				0.054
Male	181	102	79	
Female	68	29	39	
Site of tumor				<0.001
Upper	93	55	38	
Middle	83	46	37	
Low	57	30	27	
Diffuse	16	0	16	
Margin status				0.012
R0	205	99	106	
R1	35	26	9	
R2	9	6	3	
Surgical approach				<0.001
Open	144	32	112	
Laparoscopic	105	99	6	
Gastrectomy type				<0.001
Total	180	104	76	
Subtotal	27	0	27	
Distal	39	27	12	
Proximal	3	0	3	
Dissection of lymph nodes				<0.001
D1	46	12	34	
D2	203	119	84	
Complications				<0.001
No	200	117	83	
Yes	49	14	35	
TRG				<0.001
0-1	47	15	32	
2	74	28	46	
3	128	88	40	
ypTNM stage				<0.001
I	52	13	39	
II	63	29	34	
III	108	65	43	
IV	26	24	2	
Adjuvant chemotherapy				0.184
No	101	48	53	
Yes	148	83	65	
Tumor size				0.366
<5cm	117	58	59	
≥5cm	132	73	59	
Lauren histotype				0.122
Diffuse	185	92	93	
Intestinal	64	39	25	
Construction after gastrectomy				<0.001
Total/subtotal loux-en-y	199	105	94	
B-II	34	13	21	
B-I	13	13	0	
Others	3	0	3	

### Impact of TRG on Survival

**Figure 1:**
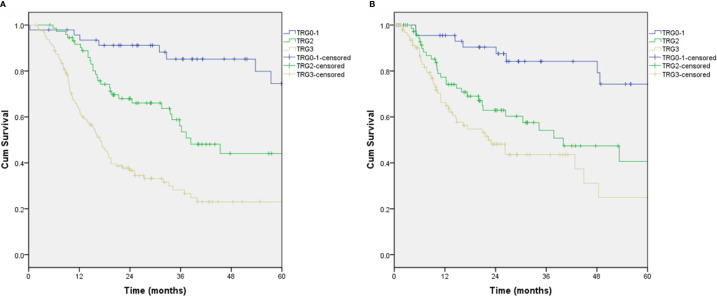
Overall survival and recurrence-free survival from TRG scores. **(A)** Overall survival, P<0.001; **(B)** Recurrence-free survival, P<0.001.

After a median follow-up of 38.8 (95% CI: 34.1–43.6) months, the overall survival rates at 3 and 5 years were 48.1% and 39.5%, respectively, in the total cohort. For patients with TRG 0-1, the 3- and 5-year survival rates were 85.2% and 74.5%, respectively, when compared to 56.1% and 44.1% with TRG 2, and 28.2% and 23.0% with TRG 3, respectively (p<0.001) (**Figure 1A**). Univariable Cox analyses revealed sex (p=0.026), margin status (p<0.001), TRG (p=0.001), ypTNM stage (p<0.001), adjuvant chemotherapy (p=0.001), tumor size (p=0.001), Lauren histotype (p=0.031), and construction after gastrectomy (p=0.032) as significant risk factors for overall survival. Multivariable Cox analyses revealed that only margin status (p=0.001) and TRG (p=0.001) were independent risk factors for overall survival (**Table 2**). Overall survival curves adjusted by multivariate models was shown in **Figure S1A**.

**Table 2:** Univariate and Multivariate Analyses for Overall Survival (n=249).

Characteristic		Univariable			Multivariable	
	N (%)	5-year OS (%)	P value	HR	95% CI	P value
Age (year)			0.876			
<65	159 (63.9)	40.2				
≥65	90 (36.1)	38.5				
Sex			0.026			0.457
Male	181 (72.7)	45.3		reference		
Female	68 (27.3)	23.5		0.859	0.576-1.282	
Country			0.706			
China	131 (52.6)	50.0				
USA	118 (47.4)	34.0				
Site of tumor			0.090			
Upper	93 (37.4)	45.5				
Middle	83 (33.3)	32.3				
Lower	57 (22.9)	50.4				
Diffuse	16 (6.4)	8.6				
Margin status			<0.001			0.036
R0	205 (82.3)	48.4		reference		
R1	35 (14.1)	0.0		0.693	0.419-1.147	0.154
R2	9 (3.6)	0.0		1.498	1.015-2.211	0.042
Surgical approach			0.099			
Open	144 (57.8)	33.1				
Laparoscopic	105 (42.2)	56.1				
Gastrectomy type			0.314			
Total	180 (72.3)	34.0				
Subtotal	27 (10.8)	50.7				
Distal	39 (15.7)	56.4				
Proximal	3 (1.2)	33.0				
Dissection of lymph nodes			0.065			
D1	46 (18.5)	22.0				
D2	203 (81.5)	45.6				
Complications			0.490			
No	625 (80.3)	39.7				
Yes	49 (19.7)	39.6				
TRG			<0.001			0.018
0-1	47 (18.9)	74.5		reference		
2	74 (29.7)	44.1		2.772	1.020-7.533	0.046
3	128 (51.4)	23.0		5.326	1.640-17.292	0.005
ypTNM stage			<0.001			0.171
I	52 (20.1)	70.7		reference		
II	63 (25.3)	43.4		1.156	0.459-2.909	0.759
III	108 (42.2)	31.2		0.891	0.290-2.741	0.841
IV	26 (10.4)	0.0		1.96	0.496-7.745	0.337
Adjuvant chemotherapy			0.027			0.251
No	101 (40.6)	27.9		reference		
Yes	148 (59.4)	47.1		0.798	0.542-1.173	
Tumor size			<0.001			0.248
<5 cm	117 (47.0)	47.1		reference		
≥5 cm	132 (53.0)	27.9		1.312	0.828-2.081	
Lauren histotype			0.031			0.356
Diffuse	185 (74.3)	34.4		reference		
Intestinal	64 (25.7)	55.6		0.797	0.492-1.291	
Construction after gastrectomy			0.032			0.190
Total/subtotal Roux-en-Y	199 (79.9)	35.1		reference		
B-II	34 (13.7)	44.7		0.999	0.582-1.716	0.998
B-I	13 (5.2)	92.3		0.129	0.018-0.947	0.044
Others	3 (1.2)	33.3		1.819	0.418-7.918	0.426

The recurrence-free survival rates at 3 and 5 years were 56.7% and 44.3%, respectively. For patients with TRG 0-1, the 3- and 5-year recurrence-free survival rates were 84.2% and 74.3%, respectively, when compared to 54.2% and 40.6% with TRG 2 and 43.6% and 24.9% with TRG 3, respectively (p<0.001) (**Figure 1B**). Univariable Cox analyses revealed country (p=0.026), margin status (p=0.049), dissection of lymph nodes (p=0.021), TRG (p<0.001), ypTNM stage (p<0.001), and tumor size (p=0.001) as significant risk factors for recurrence-free survival. Multivariable Cox analyses revealed that only TRG (p=0.007) was an independent risk factor for recurrence-free survival (**Table 3**). Recurrence-free survival curves adjusted by multivariate models was shown in **Figure S1B**.

**Table 3:** Univariate and Multivariate Analyses for Recurrence-free Survival (N=249).

Characteristic		Univariable			Multivariable	
	N (%)	5-year RFS (%)	P value	HR	95% CI	P value
Age (year)			0.518			
<65	159 (63.9)	39.5				
≥65	90 (36.1)	52.8				
Sex			0.398			
Male	181 (72.7)	47.2				
Female	68 (27.3)	33.4				
Country			0.003			0.199
China	131 (52.6)	51.0		reference		
USA	118 (47.4)	36.8		1.945	0.705-5.368	
Site of tumor			0.070			
Upper	93 (37.4)	52.6				
Middle	83 (33.3)	36.1				
Low	57 (22.9)	51.3				
Diffuse	16 (6.4)	25.9				
Margin status			0.049			0.212
R0	205 (82.3)	46.4		reference		
R1	35 (14.1)	0.0		1.321	0.419-1.147	0.570
R2	9 (3.6)	0.0		6.25	0.722-54.072	0.096
Surgical approach			0.131			
Open	144 (57.8)	42.8				
Laparoscopic	105 (42.2)	44.3				
Gastrectomy type			0.222			
Total	180 (72.3)	39.1				
Subtotal	27 (10.8)	46.4				
Distal	39 (15.7)	61.9				
Proximal	3 (1.2)	50.0				
Dissection of lymph nodes			0.021			0.066
D1	46 (18.5)	29.7		reference		
D2	203 (81.5)	47.1		0.600	0.348-1.034	
Complications			0.268			
No	625 (80.3)	45.9				
Yes	49 (19.7)	36.8				
TRG			<0.001			0.007
0-1	47 (18.9)	74.3		reference		
2	74 (29.7)	40.6		3.305	1.115-9.801	0.031
3	128 (51.4)	24.9		7.718	2.099-28.386	0.002
ypTNM stage			<0.001			0.366
I	52 (20.1)	72.4		reference		
II	63 (25.3)	39.7		5.857	0.295-129.508	0.263
III	108 (42.2)	25.5		8.512	0.418-173.242	0.164
IV	26 (10.4)	0.0		5.471	0.281-106.712	0.262
Adjuvant chemotherapy			0.088			
No	101 (40.6)	21.8				
Yes	148 (59.4)	56.3				
Tumor size			0.001			0.260
<5 cm	117 (47.0)	53.4		reference		
≥5 cm	132 (53.0)	34.9		1.368	0.793-2.361	
Lauren histotype			0.155			
Diffuse	185 (74.3)	41.5				
Intestinal	64 (25.7)	51.5				
Construction after gastrectomy			0.057			
Total/subtotal Roux-en-Y	199 (79.9)	38.4				
B-II	34 (13.7)	55.9				
B-I	13 (5.2)	79.1				
Others	3 (1.2)	50.0				

The analyses of disease-free survival and distant-metastasis-free survival yielded the similar findings. (**Figures S2A, B**)

### Factors Predicting Pathologic Response

Univariable Cox analyses revealed that country (p<0.001), cycles of neoadjuvant chemotherapy (p=0.037), regimen (oxaliplatin-based *vs* nonoxaliplatin based) (p=0.011), and regimen (epirubicin-based *vs* nonepirubicin-based) (p=0.005) were associated with TRG. Multivariable analysis revealed that only oxaliplatin-based regimen (p=0.017) was the strongest predictor of TRG (**Table 4**).

**Table 4:** Univariate and Multivariate Analysis for Predictors of TRG.

Characteristic	Univariable		Multivariable		
	P	OR	95% CI		P value
Age (<65 *vs* ≥65 yrs)	0.178				
Sex (female *vs* male)	0.167				
Country(China *vs* USA)	<0.001	0.417	0.136	1.275	0.125
Site of tumor					
upper	reference				
middle	0.522				
low	0.218				
diffuse	0.998				
Lauren histotype(diffuse *vs* intestinal)	0.071				
Cycles of neoadjuvant chemotherapy(<3 *vs* ≥3)	0.037	0.865	0.316	2.368	0.777
Regiment(Oxaliplatin based *vs*	0.011	2.889	1.212	6.885	0.017
non-Oxaliplatin based)					
Regiment(Epirubicin based *vs*	0.005	1.436	0.595	3.468	0.421
non-Epirubicin based)					

### Effects of Oxaliplatin-Based Regimen on Overall Survival and Recurrence-Free Survival

**Figure 2:**
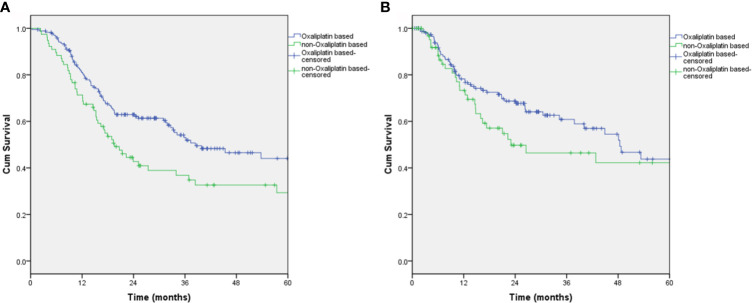
Overall survival and recurrence-free survival of patients who received the oxaliplatin-based regimen. **(A)** Overall survival, P=0.01; **(B)** Recurrence-free survival, P=0.178.

Kaplan-Meier survival estimates comparing adjuvant oxaliplatin-based regimens with nonoxaliplatin-based regimens are illustrated in **Figure 2**. The median OS of patients receiving the oxaliplatin-based regimen was significantly better than those receiving the nonoxaliplatin-based regimen (38.4 *vs* 19.5 months, respectively; p=0.01) (**Figure 2A**). There was a trend toward improving recurrence-free survival in patients receiving the oxaliplatin-based regimen; however, this trend did not reach statistical significance (48.4 *vs* 23 months, respectively; p=0.178) (**Figure 2B**).

### Subgroup Analyses by Histology All Subtype

**Figure 3:**
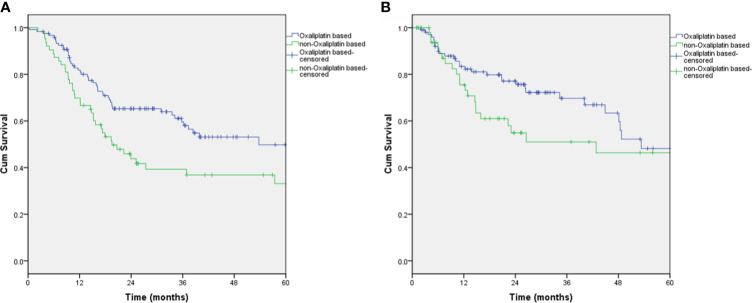
Overall survival and recurrence-free survival of non-SRCC patients who received the oxaliplatin-based regimen. **(A)** Overall survival, P=0.011; **(B)** Recurrence-free survival, P=0.14.

Among the 65 signet-ring cell carcinoma (SRCC) patients, 51 (78.5%) had received the oxaliplatin-based regimen, and 14 (21.5%) had not. When comparing with and without oxaliplatin-based SRCC patient groups, the median OS rates were 31.5 months versus 18.9 months (p=0.272), and the median RFS was 21.5 months versus 17.3 months (p=0.371), respectively. Among the 184 non-SRCC patients, 121 (65.8%) had received an oxaliplatin-based regimen, and 63 (34.2%) had not. When comparing with and without oxaliplatin-based non-SRCC patient groups, the median OS rates were 53.7 months versus 19.5 months, respectively (p=0.011) (**Figure 3A**). There was a significant improvement in the overall survival in patients who received-oxaliplatin-based regimens. The oxaliplatin-based regimen for patients with neoadjuvant chemotherapy showed a trend toward improving recurrence-free survival (**Figure 3B**); however, this result did not reach statistical significance when compared to the nonoxaliplatin-based regimen (53.3 versus 42.8 months, p=0.14, respectively).

## Discussion

The present study has demonstrated that the results of the histological-based evaluation were a good prognostic predictor for advanced gastric cancer patients receiving neoadjuvant chemotherapy. In addition, the factors predicting the histological tumor regression grading were explored.

Recently, a study suggested that TRG 1a/b is associated with improved survival (median OS>69.8 *vs* 22.8 months), but this association was not statistically significant, and a multivariate analysis was unable to confirm the predictive value of TRG. However, it should be noted that only 58 patients with neoadjuvant chemotherapy were included in this study (16). In contrast, Becker K et al. reported that TRG was an independent prognostic factor in the analysis of 480 patients with locally advanced gastric cancer treated with neoadjuvant chemotherapy followed by radical gastrectomy (17). In addition, a meta-analysis of 17 published studies also confirmed that major pathologic response is associated with a significant improvement in OS compared to no response or minor pathologic changes after neoadjuvant therapy in gastro-esophageal cancers (18). These findings were strongly supported by the results of the present study, in which multivariate survival analysis demonstrated that TRG was an independent prognostic factor for predicting worse OS.

A poorer prognosis of patients with SRCC compared to patients with non-SRCC has been identified in many reports. A French study revealed that perioperative chemotherapy provides no survival benefit in patients with gastric SRCC (19). To investigate the benefit of the oxaliplatin-based regimen, we stratified the analyzed differences in the survival rates between the SRCC and non-SRCC patient groups. Our data reveal that the oxaliplatin-based regimen failed to improve OS and RFS in patients with SRCC, indicating that the oxaliplatin-based regimen may not be the optimal choice of neoadjuvant chemotherapy for these patients.

There were several limitations in the present study. First, selected bias was inevitable in this retrospective study. Second, due to the diversity of chemotherapy regimens used in the two investigated countries and the data limited, we were unable to obtain a specific regimen (including dose and cycles) that was effective for a particular histopathological type. Despite these limitations, the present study was the first international study to explore the factors affecting TRG and to reveal that oxaliplatin-based neoadjuvant chemotherapy has potential benefits for OS in patients with nonsignet ring cell carcinoma.

In conclusion, our results suggested that TRG was an independent factor of gastric cancer treated with neoadjuvant chemotherapy plus surgery. Oxaliplatin-based neoadjuvant chemotherapy regimens improve tumor response and may benefit the OS of patients with nonsignet ring cell carcinoma.

## Data Availability Statement

The raw data supporting the conclusions of this article will be made available by the authors, without undue reservation.

## Ethics Statement

The studies involving human participants were reviewed and approved by The Institutional Review Board of Fujian Medical University Union Hospital. The patients/participants provided their written informed consent to participate in this study.

## Author Contributions

J-WX, JL, B-bX, MT, and C-MH conceived the study, analyzed the data, and drafted the manuscript. C-HZ helped critically revise the manuscript for important intellectual content. PL, J-BW, J-XL, Q-YC, L-LC, ML, R-HT, Z-NH, and J-LL helped collect data and design the study. All authors contributed to the article and approved the submitted version.

## Funding

This study was funded by the Joint Funds for the innovation of science and Technology, Fujian province (2017Y9011, 2017Y9004, 2018Y9041); the second batch of special support funds for Fujian Province innovation and entrepreneurship talents (2016B013); Construction Project of Fujian Province Minimally Invasive Medical Center (No. [2017]171); Natural Science Foundation of Fujian Province (2019J01155); Fujian provincial science and technology innovation joint fund project plan (2018Y9005); Fujian provincial health technology project (2019-ZQN-37).

## Conflict of Interest

The authors declare that the research was conducted in the absence of any commercial or financial relationships that could be construed as a potential conflict of interest.

## Publisher’s Note

All claims expressed in this article are solely those of the authors and do not necessarily represent those of their affiliated organizations, or those of the publisher, the editors and the reviewers. Any product that may be evaluated in this article, or claim that may be made by its manufacturer, is not guaranteed or endorsed by the publisher.

## Supplementary Material

Supplementary Figure 1 Survival curves adjusted by multivariate models from TRG scores. **(A)** Overall survival, P<0.001; **(B)** Recurrence-free survival, P<0.001.

Supplementary Figure 2 Disease-free survival and distant-metastasis-free survival from TRG scores. **(A)** Disease-free survival, P<0.001; **(B)** Distant-metastasis-free survival, P<0.001.
