# Supplementary figures and images for: Prognostic Value of Tumor Regression Grading in Patients Treated With Neoadjuvant Chemotherapy Plus Surgery for Gastric Cancer
Source: Front Oncol. 2021 Jul 26;11:587856. doi: 10.3389/fonc.2021.587856 (PMC8352744; doi:10.3389/fonc.2021.587856)

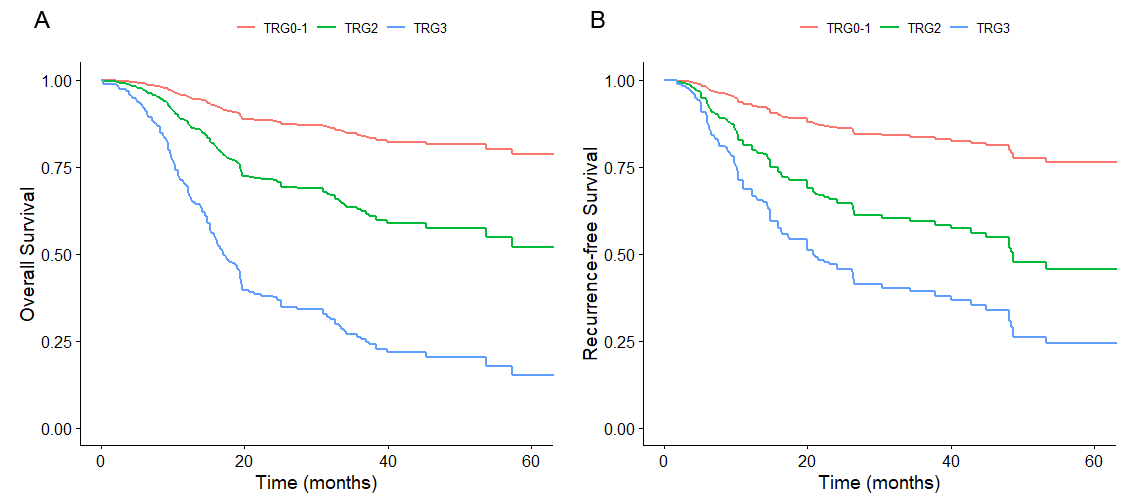

Supplement: Supplementary Figure 1 — Survival curves adjusted by multivariate models from TRG scores. (A) Overall survival, P<0.001; (B) Recurrence-free survival, P<0.001. [file Image_1.tif]

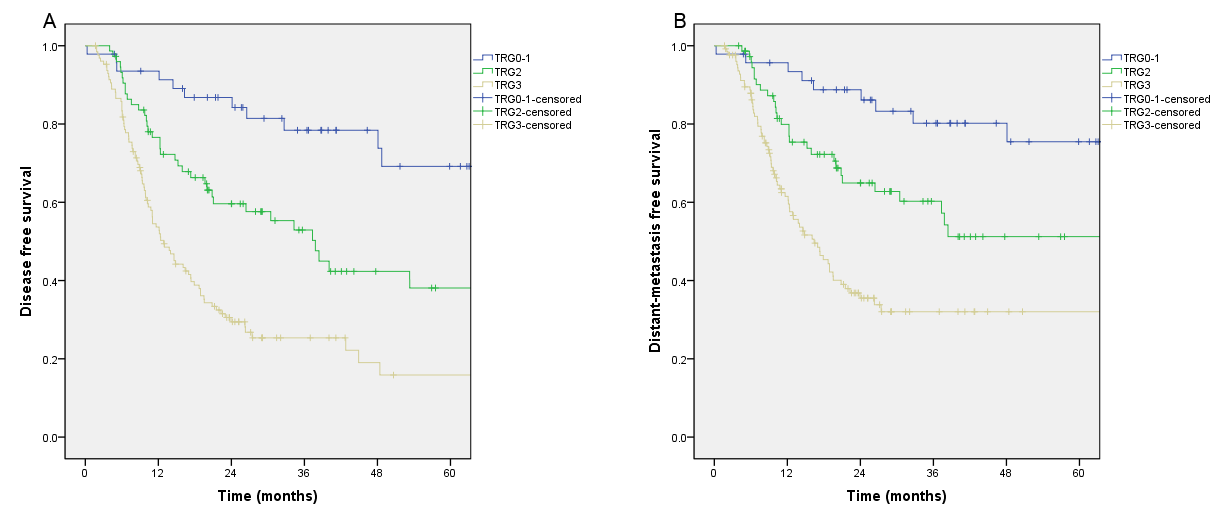

Supplement: Supplementary Figure 2 — Disease-free survival and distant-metastasis-free survival from TRG scores. (A) Disease-free survival, P<0.001; (B) Distant-metastasis-free survival, P<0.001. [file Image_2.tif]
