# Supplementary material for: Prognostic Value of Tumor Regression Grading in Patients Treated With Neoadjuvant Chemotherapy Plus Surgery for Gastric Cancer
Source: Front Oncol. 2021 Jul 26;11:587856. doi: 10.3389/fonc.2021.587856 (PMC8352744; doi:10.3389/fonc.2021.587856)
Supplement: Supplementary file 3 [file Table_1.docx]

| **Supplementary Table 1 Baseline Characteristics** | | | | |
| --- | --- | --- | --- | --- |
|  | TRG | | | |
| Characteristic | 0-1 (n=47) | 2 (n=74) | 3 (n=128) | p value |
| Age(year) |  |  |  | 0.284 |
| <65 | 26 | 46 | 87 |  |
| ≥65 | 21 | 28 | 41 |  |
| Sex |  |  |  | 0.329 |
| Male | 9 | 20 | 39 |  |
| Female | 38 | 54 | 89 |  |
| Site of tumor |  |  |  | 0.218 |
| Upper | 18 | 28 | 47 |  |
| Middle | 13 | 23 | 47 |  |
| Low | 16 | 17 | 24 |  |
| Diffuse | 0 | 6 | 10 |  |
| Margin status |  |  |  | <0.001 |
| R0 | 45 | 70 | 90 |  |
| R1 | 1 | 4 | 30 |  |
| R2 | 1 | 0 | 8 |  |
| Surgical approach |  |  |  | 0.066 |
| Open | 30 | 49 | 65 |  |
| Laparoscopic | 17 | 25 | 63 |  |
| Gastrectomy type |  |  |  | 0.037 |
| Total | 27 | 51 | 102 |  |
| Subtotal | 10 | 7 | 10 |  |
| Distal | 9 | 14 | 16 |  |
| Proximal | 1 | 2 | 0 |  |
| Dissection of lymph nodes |  |  |  | 0.023 |
| D1 | 14 | 16 | 16 |  |
| D2 | 33 | 58 | 112 |  |
| Complications |  |  |  | 0.132 |
| No | 35 | 65 | 100 |  |
| Yes | 12 | 9 | 28 |  |
| ypTNM stage |  |  |  | <0.001 |
| I | 39 | 13 | 0 |  |
| II | 8 | 46 | 9 |  |
| III | 0 | 15 | 93 |  |
| IV | 0 | 0 | 26 |  |
| Adjuvant chemotherapy |  |  |  | 0.049 |
| No | 12 | 30 | 59 |  |
| Yes | 35 | 44 | 69 |  |
| Tumor size |  |  |  | <0.001 |
| <5cm | 37 | 45 | 35 |  |
| ≥5cm | 10 | 29 | 93 |  |
| Lauren histotype |  |  |  | 0.123 |
| Diffuse | 30 | 54 | 101 |  |
| Intestinal | 17 | 20 | 27 |  |
| Construction after gastrectomy |  |  |  | 0.118 |
| Total/subtotal loux-en-y | 34 | 57 | 108 |  |
| B-II | 9 | 8 | 17 |  |
| B-I | 3 | 7 | 3 |  |
| Others | 1 | 2 | 0 |  |
